# Sheep scab spatial distribution: the roles of transmission pathways

**DOI:** 10.1186/s13071-021-04850-y

**Published:** 2021-06-29

**Authors:** Emily Joanne Nixon, Ellen Brooks-Pollock, Richard Wall

**Affiliations:** 1 School of Biological Sciences, University of Bristol, Bristol, BS8 1TQ UK; 2 Veterinary Public Health, Bristol Veterinary School, University of Bristol, Bristol, BS40 5EZ UK; 3 NIHR Health Protection Research Unit in Behavioural Science and Evaluation, University of Bristol, Bristol, UK

**Keywords:** Disease, Epidemiology, Infection, Mange, Model, Movement, Network, *Psoroptes*, Parasites

## Abstract

**Background:**

Ovine psoroptic mange (sheep scab) is a highly pathogenic contagious infection caused by the mite *Psoroptes ovis*. Following 21 years in which scab was eradicated in the UK, it was inadvertently reintroduced in 1972 and, despite the implementation of a range of control methods, its prevalence increased steadily thereafter. Recent reports of resistance to macrocyclic lactone treatments may further exacerbate control problems. A better understanding of the factors that facilitate its transmission are required to allow improved management of this disease. Transmission of infection occurs within and between contiguous sheep farms* via* infected sheep-to-sheep or sheep–environment contact and through long-distance movements of infected sheep, such as through markets.

**Methods:**

A stochastic metapopulation model was used to investigate the impact of different transmission routes on the spatial pattern of outbreaks. A range of model scenarios were considered following the initial infection of a cluster of highly connected contiguous farms.

**Results:**

Scab spreads between clusters of neighbouring contiguous farms after introduction but when long-distance movements are excluded, infection then self-limits spatially at boundaries where farm connectivity is low. Inclusion of long-distance movements is required to generate the national patterns of disease spread observed.

**Conclusions:**

Preventing the movement of scab infested sheep through sales and markets is essential for any national management programme. If effective movement control can be implemented, regional control in geographic areas where farm densities are high would allow more focussed cost-effective scab management.

**Graphical Abstract:**

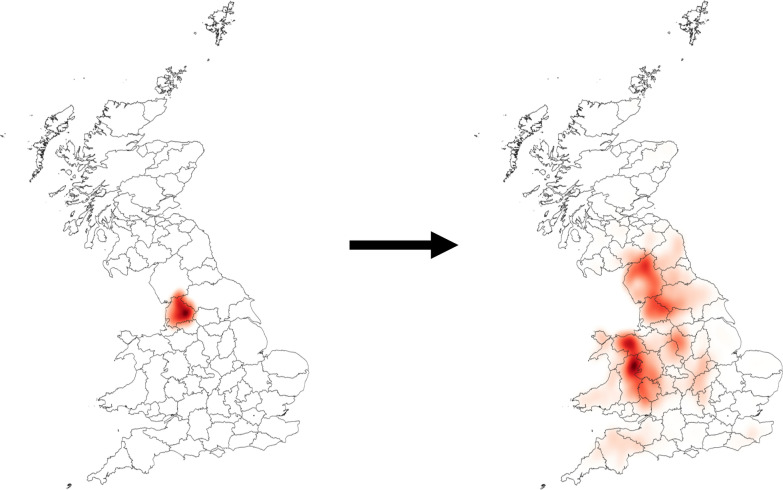

## Background

Ovine psoroptic mange (sheep scab) is the result of a hypersensitivity response to the faecal material of the parasitic mite, *Psoroptes ovis* [1]. This highly pathogenic and infectious ectoparasitic condition impacts sheep husbandry systems in many parts of the world [2]. In the UK, sheep scab was eradicated in 1952 [3]; however, it was inadvertently reintroduced in 1972 on imported animals [4]. Although movement restrictions and a series of compulsory regional and national acaricide treatment programmes were imposed from 1973 to 1992, sheep scab persisted throughout the UK during this period [5]. Deregulation and the loss of statutory control in 1992 resulted in a rapid increase in incidence; today, scab affects about 9% of flocks annually in the UK [6, 7], with a particularly high incidence in Wales (15.8%) [8], Scotland (14%) and northern England (11%) [6].

Economic losses resulting from scab are due to pruritus and excoriation, leading to wool loss [9], weight loss [10], reproductive losses [11] and, ultimately, the mortality of infested hosts [12]. These losses, along with the associated costs of treatment, food and labour, are estimated to cost the sheep industry in the UK approximately GBP 78–202 million per year [13]. Hence, sheep scab represents a significant economic and animal welfare issue.

Organophosphate plunge dips and injectable macrocyclic lactones are the only licensed products for scab currently available, and these are used as both prophylaxis and therapeutic treatment. Farmers have flexibility in the choice of these treatments, although prophylaxis is optional while the treatment of confirmed outbreaks is compulsory [14] and cases in Scotland must be reported [15]. However, in light of recently reported resistance in *P. ovis* to moxidectin [16], ivermectin and doramectin [17], it has been suggested that more coordinated treatment programmes between farms and regions are needed to prevent the spread of resistance and to reduce the incidence of scab before it becomes still more extensive [18]. In addition, routine use of diagnostic tests would reduce unnecessary treatment use and help to prevent undetected transmission. Although scab has been traditionally diagnosed* via* wool and skin scrapings, the success rate of this diagnostic method has been reported to be as low as 18% [19]; consequently, the enzyme-linked-immunosorbent assay (ELISA), a diagnostic tool for sheep scab [20] with sensitivity of 98.2% and specificity 96.5% [21], is currently the most effective method for diagnosis.

Scab is transmitted between neighbouring farms through physical contact between sheep at farm boundaries, in areas of common grazing where sheep from different farms share pasture, or through environmental contamination [22]. Off-host mites can persist in a contaminated environment for up to 20 days [23]. Having neighbours with scab and the use of common grazing have been shown to increase the risk of scab infection by tenfold [24]. Hence, coordinated treatment between contiguous farms within regions is likely to result in more effective control. Sheep scab can also be introduced into a naïve flock through animals bought from markets [22], although the relative risk from this transmission route remains unquantified.

One approach to assessing the role of various transmission pathways and their contribution to the observed national pattern of scab incidence is through the use of networked metapopulation models. Metapopulation models, first used in ecology for the investigation of population dynamics [25], have been applied for modelling disease transmission, including veterinary diseases such as equine influenza [26]. They have also been used along with network theory and dynamic epidemic transmission models to explore local and global disease dynamics [27].

A spatially explicit model for sheep scab has been developed [28, 29]. The model uses the physical coordinates of all sheep holdings in the UK and the number of ewes at each holding, as well as official sheep movement data, to produce spatially explicit stochastic simulations of sheep scab incidence and distribution. In the model, transmission occurs directly between sheep within sheep holdings and between contiguous sheep holdings,* via* the contaminated environment (fencing and handling equipment) and through the long-distance movements of sheep to markets. However, the relative importance of these two transmission routes in explaining the spatial patterns of sheep scab has not yet been investigated. The aim of the study reported here, therefore, was to examine the relative importance of transmission between contiguous sheep holdings or long-distance sheep movement on the spatial dynamics of sheep scab, given the underlying distribution of sheep holdings in the UK.

## Methods

### Model description

A stochastic networked metapopulation model for sheep scab [29] was used for the simulations described herein. The model is written in the programming language R v.3.6.3 [30] and is freely available [28]. Within the model, georeferenced sheep holdings are subpopulations, within which the dynamics of scab are modelled using an epidemic compartmental model first developed by Ronald Ross [31] and now used extensively for a variety of infectious diseases [32]. Sheep within a holding are classified as susceptible (S), infected (I) and carrier (C) and move between these three disease states at specified rates. Infected and carrier sheep are both infectious, but the rate is lower for carriers. An additional compartment representing the environment, *e*, exerts an infectious pressure on susceptible sheep, determining the number that become infected. The infectious pressure in *e* is contingent on the shedding of *P. ovis* mites from infectious (I and C) sheep within a sheep holding.

Transmission of scab between sheep holdings can occur through increased infectious pressure risk in the environment (*e*) of contiguous sheep holdings, which are assumed to be holdings that are ≤ 2 km apart. This assumption is based on the sum of the radius of an average farm [33] (assuming it is circular) and the radius of an adjacent holding, as described in [29]. The impact of the environmental pressure from contiguous sheep holdings is applied when the rate of change of the environmental infectious pressure over time is calculated. The impact a contiguous sheep holding has on the environmental infectious pressure of a particular holding is scaled by the distance between the two holdings and cannot be greater than the impact attributed to sheep within the particular holding.

Transmission of scab between sheep holdings can also occur* via* the long-distance movements of infectious sheep, which can be specified in the model as “events” that are executed when the simulation (in continuous time) reaches an event’s associated timestep. At each timestep of the model, the specified number of individuals that are to be moved are sampled at random from across all disease compartments in the source node and then transferred to their corresponding disease compartment in the destination node. All transitions are modelled as continuous-time discrete Markov chains using the direct method [34] as fully described in [35, 36].

Most model parameters were derived from published data [29]. However, the daily contribution to environmental pressure per infected individual (*α*), the decay rate of the environmental infectious pressure (*β*) and the indirect transmission rate from the environmental compartment* j* to susceptible sheep in farm* i* (*υj*) were derived by fitting the model to weekly and yearly scab incidence cases from 1973 to 1992 using sequential Monte Carlo approximate Bayesian Computation methods as described in [29]. The values for these estimated parameters were selected at the upper ranges of the posterior distribution for the two transmission rates (*υj * = 6 × 10^4^,* α * = 1 × 10^2^) and the lower range of the distribution for the disease decay rate (*β* = 4 × 10^2^), in order to allow for transmission patterns across the network to be observed.

### Sheep holding and movement data

Agricultural survey data and sheep movement data for 2010 for the UK were provided by the Animal and Plant Health Agency (APHA). These data were used to estimate the number of sheep at each sheep holding at the start of the model simulations [29] and to create the movement events for the model simulations. The number of sheep at permanent animal residences (farms) were estimated by reconciling the survey and movement data as described in [29]. The Euclidean distances between the centres of all farms were calculated in the R statistical software v.3.6.3 [30] using the easting and northing for each farm. For each farm, the distance to the nearest farm was identified, as were all distances to other farms of ≤ 2 km.

Spatial heatmaps were produced in QGIS version 3.4.15 [37]. First, the density of sheep farms was weighted by the number of contiguous farms (Kernel density estimate with 1-km^2^ grids, quartic kernel shape, a search radius of 10 km and colour shades determined by quantile). In order to identify which farms were in the areas of highest density and connectivity, a shapefile was drawn over the darkest red regions from Fig. 2 and the “Clip” tool in QGIS was used to select farms which fall within these areas. A further heatmap was made showing the density of sheep farms weighted by the number of movements per year associated with each farm using the heatmap style in the layer properties.

### Scenario analysis

The transmission and spatial distribution of scab was investigated under different scenarios. Assuming at the start that no scab was present, sheep scab was then introduced to 50 randomly selected farms in areas of high farm density and connectivity, first into a cluster in the North West of England (Lancashire) or into a similar cluster in the South West of England (Devon). The farms were selected at random. It was assumed that all sheep in each infected farm were infected. The model was then used to examine the expected spread of scab in the 12 months following introduction, in the presence or absence of long-distance movements. Since the model is stochastic, each scenario was repeated 60 times. The results for each scenario were displayed using the heatmap style in the layer properties using QGIS version 3.4.15 [37].

In order to test any uncertainty around the assumption that transmission can occur between contiguous sheep holdings located ≤ 2 km apart, further simulations of the model were run using a 4-km cut-off with the same Lancashire farms that were initially infected in the 2-km scenarios. This test included four simulations where long-distance movements were included and four in which they were not. The difference in the number of farms infected throughout the year between the 2-km and 4-km scenarios was calculated and, as with the 2-km scenarios, the results from the 4-km scenarios were displayed spatially using the heatmap style in the layer properties using QGIS version 3.4.15 [37].

## Results

**Fig. 1:**
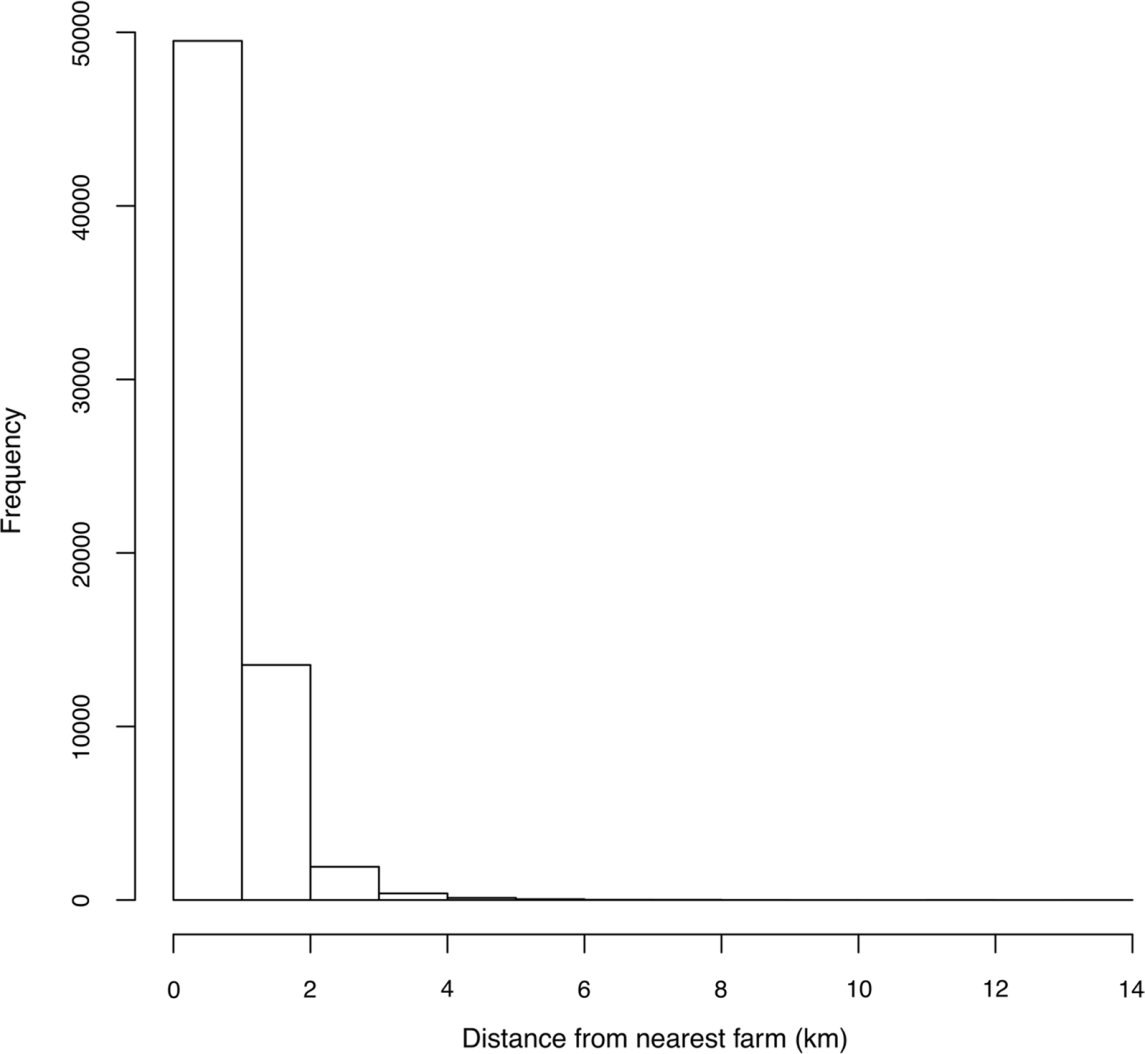
The distribution of distances to nearest permanent animal residence for each permanent animal residence. One distance was not included in the histogram (24 km) because it was considered to be an outlier

There were 111,177 spatial coordinates of sheep holdings in the model, of which 68,620 were farms (permanent sheep residences) where all 37,191,725 sheep in the model were located at the start of the simulations. The remaining sheep holdings were locations where sheep are temporarily held, such as markets and other animal gatherings. The majority of farms were contiguous with < 20 other farms, 96% of farms were within 2 km of their nearest neighbour (Fig. 1), with four contiguous farms being the mode, and 2395 farms were not contiguous with any other farms. The modal number of contiguous farms in Scotland (3) was lower than that for England (4) and Wales (9), and the majority of farms in Scotland have < 13 contiguous neighbours. However, unlike England and Wales there are some farms in Scotland that have > 40 contiguous neighbours.

**Fig. 2:**
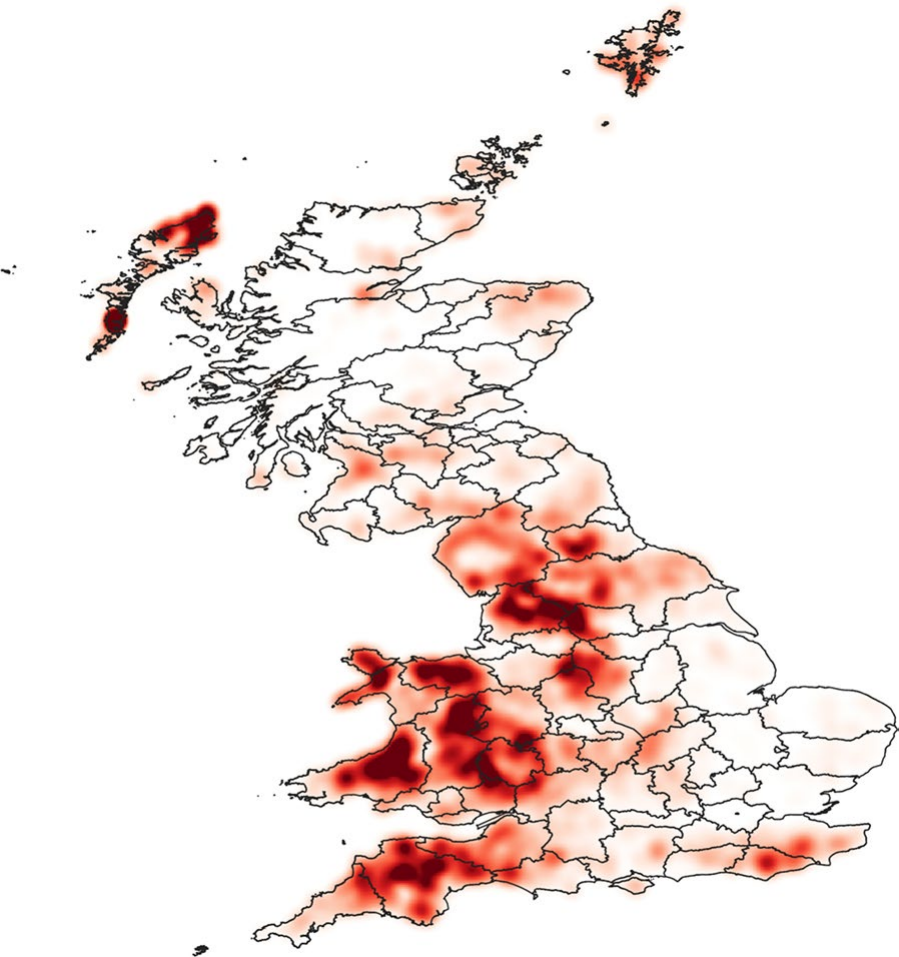
The density of sheep farms in the UK weighted by the number of contiguous farms. The darker the shading, the higher the density of farms and the higher the number of connections. Data on the locations of farms and number of sheep per farm were provided by the Animal and Plant Health Agency, and kernel density methods were used with search radius of 10 km, a quartic kernel shape with 1-km^2^ pixel grids and colour shades determined by quantile. Only sheep holdings considered to be permanent animal residences were included

Areas where a relatively high density of farms had a relatively high number of contiguous neighbours are referred to here as ‘contiguous clusters’ (indicated by the darkest red regions in Fig. 2). Regions in the UK which had a large number of contiguous clusters are Wales, South West England, some areas of northern England and some Scottish islands (Fig. 2). Farms in the contiguous clusters consistently had higher numbers of contiguous farms (mean = 23, median = 18, mode = 17, interquartile range [QR] = 13–23) than those outside of the contiguous clusters (mean = 10, median = 9, mode = 5, IQR = 5–13).

**Fig. 3:**
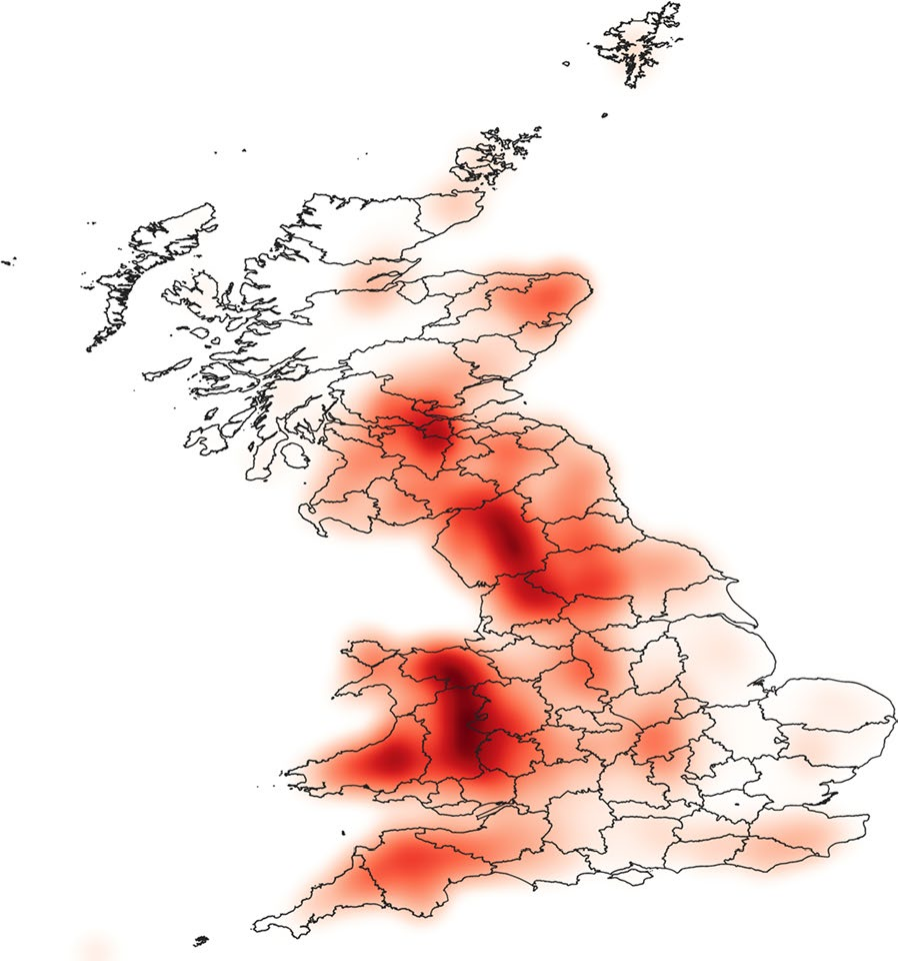
The density of sheep farms in the UK weighted by frequency of associated sheep movements. The darker the shading, the higher the density of sheep farms and the higher the number of associated movements. Data on the locations of farms, the number of sheep per farm and sheep movements were provided by the Animal and Plant Health Agency. Only sheep holdings considered to be permanent animal residences were included

Farms most commonly had one recorded sheep flock movement in a 1-year period, but on average they had ten associated movements (median = 3, IQR = 1–12). Most contiguous clusters correspond with areas associated with relatively high numbers of sheep movements (Fig. 3). The exception to this was the contiguous clusters in the Scottish islands which do not have a relatively high number of movements associated with them. Conversely, in southern Scotland, there is an area associated with relatively large numbers of animal movements (Fig. 3) that does not have a relatively high density of contiguous clusters (Fig. 2).

**Fig. 4:**
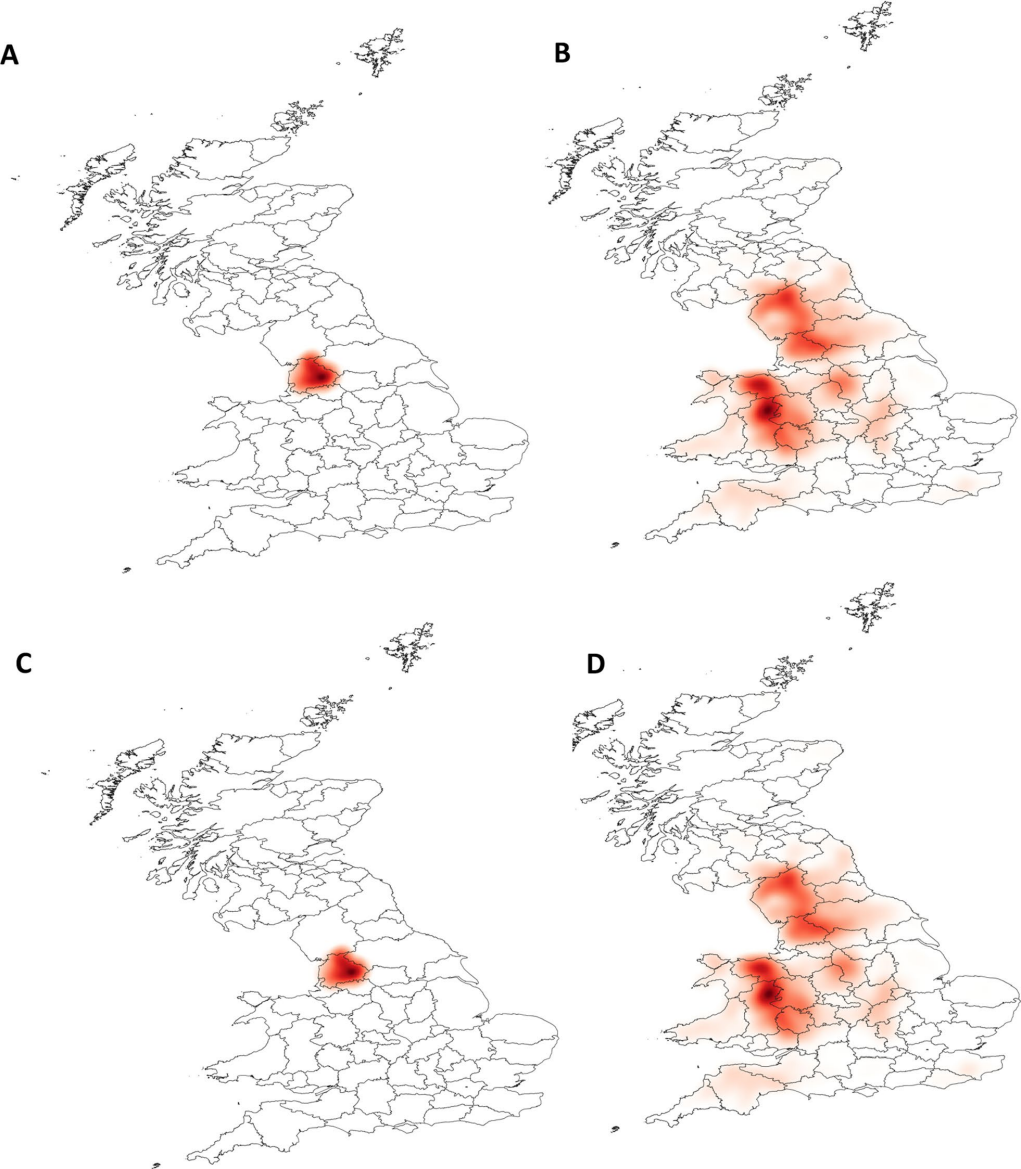
The density of infected sheep holdings following the simulated introduction of sheep scab into Lancashire. The darker the shading, the higher the density of sheep holdings which were infected in a 1-year period across 60 model simulations (**a**, **b**) and across 4 model simulations (**c**, **d**). Sheep holdings were considered to be contiguous when located ≤ 2 km apart (**a**, **b**) or ≤ 4 km apart (**c**, **d**). Sheep movements were included in the model simulations in **b** and **d** but not in the simulations for **a** and **c**

**Fig. 5:**
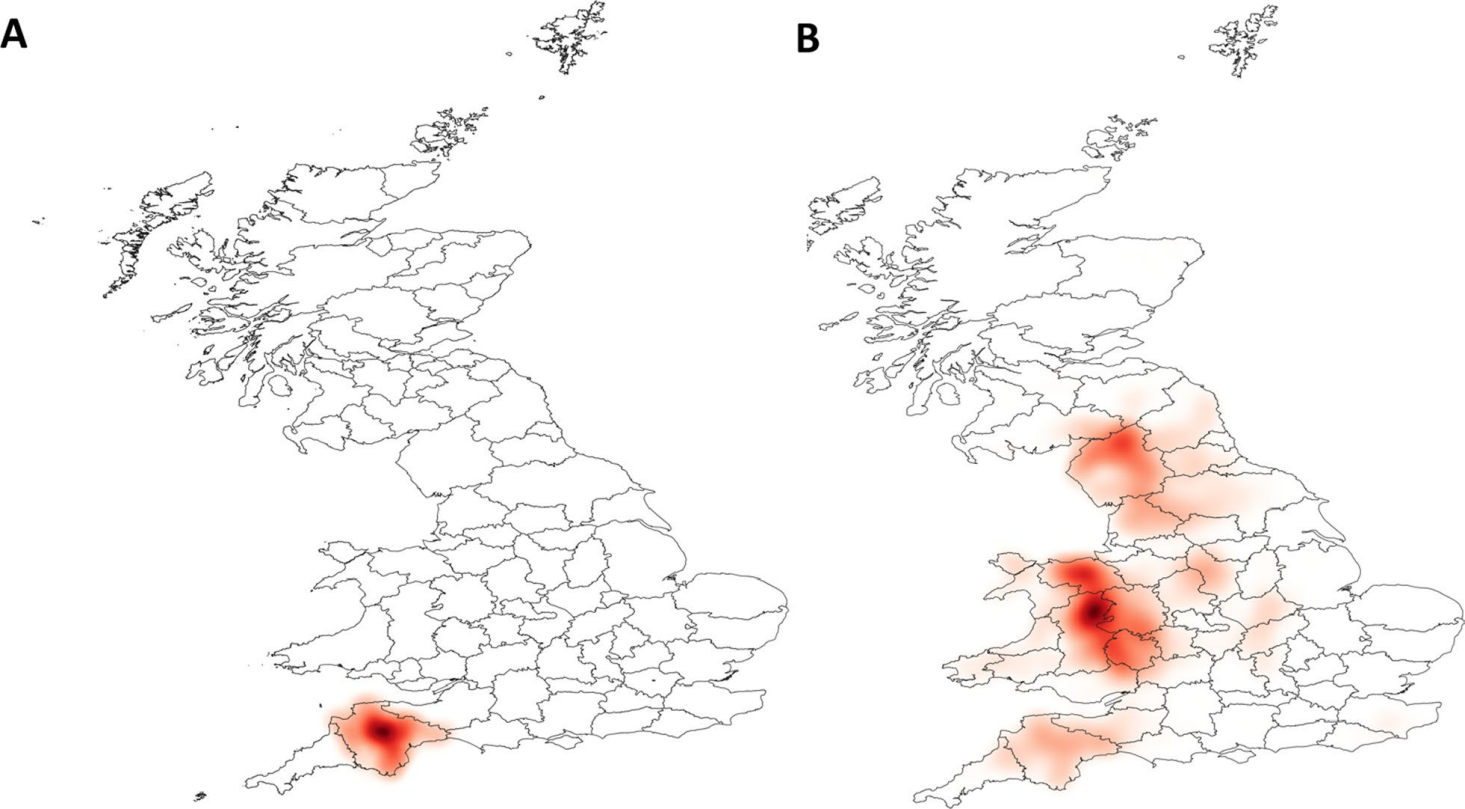
The density of infected sheep holdings following the simulated introduction of sheep scab into Devon. The darker the shading, the higher the density of sheep farms which were infected in a 1-year period across 60 simulations. **a** Sheep movements were not included in the model, **b** sheep movements were included in the model

Upon the introduction of infection into the two clusters (North West England or South West England) that contain a high density of contiguous farms and have a large number of movements, similar spatial patterns of transmission were observed (Figs. 4a, b, 5). When sheep movements were included in the model, scab spread from the clusters where they had been introduced across the whole of the UK, with a particular focus on other clusters of highly contiguous farms. However, when long-distance movements were not included there was some spread within the cluster where disease had been introduced but no further spread of scab outside of those areas.

Doubling the distance at which it was assumed transmission could occur between contiguous sheep holdings, from 2 to 4 km, led to more infected holdings (fivefold more when there were no long-distance movements and fourfold more when long-distance movements were included). However, the same spatial patterns were seen, with the disease not spreading further than the contiguous cluster in which it was introduced (Fig. 4c, d).

## Discussion

This analysis of the spatial distribution of farms identified geographical areas of the UK where there are a relatively high density of farms, each with a relatively high number of contiguous farms (described here as contiguous clusters). These generally are also geographical areas associated with relatively high numbers of sheep movements. Previous work used sheep scab outbreak data from January 1998 to December 2007 in a simple risk model, along with elevation, precipitation, temperature and sheep density, to identify specific regions as high-risk areas for sheep scab [35]. The analysis by Rose et al. [7] found that sheep density was the most important factor in explaining the spatial patterns of scab outbreaks, and the outbreak data presented in their study match closely the spatial distribution of contiguous clusters presented here, particularly for England and Wales, thereby providing good validation for the approach. However, the data presented by Rose et al. [7] indicated a slightly higher distribution of scab in Scotland than predicted in our simulations, and these authors also suggested that the importance of sheep density in explaining sheep scab risk might be lower in upland areas, where common grazing and climatic data may be more influential in explaining scab risk. Hence, the drivers of risk of sheep scab in upland areas of Scotland may differ from those in the rest of the UK, an unknown which requires further investigation.

This study highlights the importance of sheep movements in explaining the spatial epidemiology of sheep scab. The key finding is that where no long-distance movements of infected animals is permitted, scab incidence becomes restricted and localised in geographically distinct areas. This finding allows new approaches to national scab management to be considered. Preventing the long-distance movement of infected animals could be achieved by either restricting movement altogether or, more plausibly, by ensuring that any animals moved are free of scab. This could be achieved either by prophylactic use of licensed scab treatment products, or, to prevent unnecessary treatment use,* via* reactive treatment for flocks following use of a diagnostic tool, with the sheep scab ELISA being the most effective choice [20].

Future control attempts should prioritise restricting the movement of potentially infested sheep through sales and markets. If this could be achieved, targeted scab management in localised cluster areas (high farm density and with high connectedness) could be the most cost-effective approach, allowing resources to be focussed on specific regions. Previously, regional control programmes have generally been focussed within state or geopolitical (county or country) borders; an example of this approach is the sheep scab eradication programme in Dartmoor [38], Scotland or Wales [39]. However, our simulations suggest that such approaches are not likely to be effective. A more effective approach will be to identify areas of high inter-farm connectivity, and these may well cross county and country borders. Not all areas of high inter-farm connectivity may have a high prevalence of scab; however regular monitoring of scab status within these areas could be important since if scab is introduced into one farm, then it is likely to spread rapidly to the other farms. Successful implementation of these recommendations would require the cooperation of local government, farmers and veterinarians across regions. The recommendations should be implemented alongside research into the economics of sheep scab and farmer behaviour [40] to ensure that all stakeholders are motivated to participate.

It is possible that the importance of movements could be overestimated here since the model assumes that farms that do not use common grazing and that they need to be within a 2 km radius of each other to allow neighbour-to-neighbour transmission* via* the environment. These assumptions were based on the known average farm size. However, sheep farms do vary greatly in size and so there are likely to be farms in the model that should be connected but are not, and* vice versa*. Given the number of farms included, it was anticipated that such variation in farm size would be subsumed within the model, but clearly the greater the distance over which transmission by contact is possible, the more quickly scab will spread, as seen in our study when the radius was increased to 4 km. However, even in the scenarios where farms within 4 km of each other were able to transmit disease, although there were a higher number of cases, the same spatial patterns were seen, with these cases occurring in the areas of high farm density in which they were introduced when long-distance movements were not included. Therefore, this finding reinforces the conclusion about the effectiveness of focussing scab management on areas of high farm density and connectedness.

The unit of study here is the farm, and once scab is introduced onto a farm all animals were presumed to be infected; a comparative study of networked metapopulation modelling approaches for diseases has found that not including individual hosts may overestimate the spatial spread and aggregate growth of the epidemic in the model, as well as the degree of spatial synchrony and the peak number of cases [41]. However, including sheep as individuals in the model used here would increase the computational load unsustainably.

Where suitable data on sheep holding location and sheep movement are available, the model used here can be used to investigate control methods for sheep scab in areas outside of the UK. In addition, the model could be adapted and used to investigate other ectoparasite infections, such as sarcoptic mange, although this again would be contingent on suitable epidemiological data. Depending on the ectoparasite, the model may also need to be expanded to include transmission routes between hosts, or from wildlife reservoirs. These transmission routes were not explored here since they are not considered to be epidemiologically significant for *Psoroptic* mange [42]*,* even though *P. ovis* is not considered to be host specific [43]. Transmission of *P. ovis* between host species is thought to be a rare occurrence in the field and requires enforced close contact, as seen in a potential *P. ovis* transmission event between rabbits and bighorn sheep in North America held in captivity together at a game farm [44].

## Conclusions

Neighbour-to-neighbour contact allows transmission in areas of high farm density, but long-distance animal movements probably drive the national transmission pattern observed. There are two implications: future control attempts should focus on the movement of potentially infested sheep through sales and markets and, if effective movement control can be implemented, regional control in isolated geographic areas where farm densities are high should be an achievable approach to effective local scab management.

## Data Availability

The individual farm and animal movement data that support the findings of this study were provided by the Animal and Plant Health Agency of the UK Government, but restrictions apply to the availability of these data, which were used under license for the current study, and so are not publicly available from the authors.
